# Technical aspects of a new approach to intraoperative pelvic neuromonitoring during robotic rectal surgery

**DOI:** 10.1038/s41598-023-41859-y

**Published:** 2023-10-11

**Authors:** Ramona Schuler, Christoph Marquardt, Georgi Kalev, Andreas Langer, Marko Konschake, Thomas Schiedeck, Julia Bandura, Matthias Goos

**Affiliations:** 1 Research and Development, Dr. Langer Medical GmbH, Waldkirch, Germany; 2 Institute of Biomedical Engineering and Informatics, TU Ilmenau, Ilmenau, Germany; 3 Department of General, Visceral, Thoracic and Pediatric Surgery, Ludwigsburg Hospital, Ludwigsburg, Germany; 4 Department of Anatomy, Histology and Embryology, Institute of Clinical and Functional Anatomy, Medical University of Innsbruck (MUI), Innsbruck, Austria; 5 Department of General and Visceral Surgery, Helios Hospital Müllheim, Heliosweg 1, 79379 Müllheim, Germany

**Keywords:** Adaptive clinical trial, Translational research, Colon, Rectum, Colon cancer, Rectal cancer, Enteric nervous system, Muscle contraction

## Abstract

It has been found that rectal surgery still leads to high rates of postoperative urinary, fecal, or sexual dysfunction, which is why nerve-sparing surgery has gained increasing importance. To improve functional outcomes, techniques to preserve pelvic autonomic nerves by identifying anatomic landmarks and implementing intraoperative neuromonitoring methods have been investigated. The objective of this study was to transfer a new approach to intraoperative pelvic neuromonitoring based on bioimpedance measurement to a clinical setting. Thirty patients (16 male, 14 female) involved in a prospective clinical investigation (German Clinical Trials Register DRKS00017437, date of first registration 31/03/2020) underwent nerve-sparing rectal surgery using a new approach to intraoperative pelvic neuromonitoring based on direct nerve stimulation and impedance measurement on target organs. Clinical feasibility of the method was outlined in 93.3% of the cases. Smooth muscle contraction of the urinary bladder and/ or the rectum in response to direct stimulation of innervating functional nerves correlated with a change in tissue impedance compared with the pre-contraction state. The mean amplitude (Amax) of positive signal responses was Amax = 3.8%, negative signal responses from a control tissue portion with no stimulation-induced impedance change had an amplitude variation of 0.4% on average. The amplitudes of positive and negative signal responses differed significantly (statistical analysis using two-sided t-test), allowing the nerves to be identified and preserved. The results indicate a reliable identification of pelvic autonomic nerves during rectal surgery.

## Introduction

The treatment of rectal cancer has undergone huge evolution in recent decades as a result of improved surgical techniques such as the introduction of the total mesorectal excision (TME) in a laparoscopic or robotic approach. Additional factors considered to improve the prognosis for the patient significantly include multimodal therapy, high-resolution imaging for diagnostics, intraoperative fluorescence imaging, and an increased focus on the functional outcome. Nevertheless, the functional and surgical outcome depends on several risk factors like low tumor location, difficult anatomical situations (narrow male pelvis, obesity), aggressive preoperative radiotherapy, as well as implementation and feasibility of nerve-sparing surgery^1^. As the rates of postoperative urinary, fecal, or sexual dysfunction after low anterior resections are high^2–5^, techniques to preserve pelvic autonomic nerves by identifying anatomic landmarks and implementing intraoperative neuromonitoring methods have been investigated^2–5^.

Urinary dysfunction can manifest itself in a variety of ways such as urge, overflow, or stress incontinence and is accompanied by severe impairment and reduction of the quality of life for the patients. Lange et al. reported a rate of 38% of patients experiencing urinary dysfunction after rectal cancer treatment, with 72% having normal preoperative function^6^. Kauff et al. investigated urogenital dysfunction rates in a two-year clinical follow-up study after TME including minor to major disturbances. It was found that the majority of the 85 patients suffered from new-onset urogenital dysfunction, with 34% having mild and 1% having severe urinary dysfunction, while 72% of patients had impaired sexual function^3^. One-third of 52 patients who underwent TME for rectal cancer experienced fecal incontinence after a follow-up period of 2 years^4^.

A known reason for these urinary, fecal, or sexual disorders after rectal cancer treatment is surgery-related pelvic nerve injury. Even though possible previous damage to the nerves by radiation or traction is disregarded, the challenge for the surgeon is to differentiate and protect the course of the nerves by means of a layer-appropriate preparation. This can be achieved by pelvic neuromonitoring, which can have a distinct advantage in identifying the pelvic nerves and therefore preventing postoperative autonomic nerve dysfunction^3–5,7^.

The neuroanatomy of the pelvic region comprises autonomic fibers both sympathetic and parasympathetic as well as somatic fibers. In existing literature, the origin of sympathetic innervation is explained to be in the intermesenteric plexus located preaortally. This plexus derives its nerve fibers from segments Th10 to L2 of the lower thoracic and upper lumbar spinal cord^8^. Furthermore, the superior and inferior mesenteric plexuses are noted to initiate from the L3 to S1 segments of the lower lumbar and upper sacral spinal cord, converging into the superior hypogastric plexus. This plexus is situated below the point of aortic bifurcation and subsequently divides into hypogastric nerves^9–11^. These hypogastric nerves can manifest in various forms, ranging from a straightforward nerve cord to a more intricate plexus^10^. They are located in, around, and below the iliac arteries, within the retroperitoneal adipose tissue. From this location, nerve fibers extend towards the rectum and sigmoid colon. These fibers are attached to the surface of the mesorectum and then penetrate into the volume of mesorectal adipose tissue^8,12^.

The hypogastric nerves and the parasympathetic pelvic splanchnic nerves (originating from S2 to S4) together form the inferior hypogastric plexus^8,11^. This plexus holds significant importance concerning intraoperative pelvic neuromonitoring. In males, the hypogastric nerve joins the plexus at the point where the ureter meets the vas deferens. Furthermore, the inferior hypogastric plexus is formed by the sympathetic splanchnic sacral nerves, which emanate primarily from sacral segments, predominantly S2. The inferior hypogastric plexus takes on a triangular configuration, positioned on the mesorectal fascia within male anatomy. In females, its location varies more ventrally, situated either at the level of the uterosacral ligament and parametria, contingent on the woman's age. Nerves within the inferior hypogastric plexus then extend in two directions. On one side, they form lateral connections directly to the rectum. On the other side, numerous nerve fibers branch out ventrally, targeting organs such as the ureter, urinary bladder, seminal vesicles, vas deferens, penis, and prostate in males^8,11,13^.

Segments of the urethra that are in proximity to the bladder receive their supply from the inferior hypogastric plexus. The sympathetic components of the inferior hypogastric plexus, for instance, play a role in facilitating the filling of the urinary bladder, whereas the parasympathetic fibers are responsible for opening the urethra and initiating the process of micturition^10^. Emerging from the prostate, a cluster of nerves, along with blood vessels, extends to the cavernous bodies of the penis, constituting the cavernous nerve. Within the female pelvic region, nerve fibers are positioned laterally from the uterus and vagina, providing innervation to the urethra and urinary bladder^8,9,11,13^. Somatic innervation within the pelvis is established through the pudendal nerve and the coccygeal plexus, both of which supply the pelvic floor muscles and confer pelvic floor sensibility. The pudendal nerve takes origin from the sacral plexus, derived from segments S2-S4. As the pudendal nerve progresses, nerve fibers diverge to reach the external anal sphincter, the anoderm, and the perianal skin^2,8–12,14^.

The intricate nature and distinct arrangement of nerves within the pelvis arise from several contributing factors. These encompass variances between sexes, degenerative alterations in nerve branches, and the intricate, multi-layered composition of the fascia structure. For instance, the parietal pelvic fascia, which comprises both an inner and an outer lamella encompassing the hypogastric nerves. The quantity of adipose tissue situated between these lamellae varies based on individual characteristics^2,8^. Consequently, disparities in neuroanatomy between individuals exist, presenting a challenge for intraoperative neuromonitoring. Nerve identification with intraoperative neuromonitoring is based on the principle of direct stimulation of nerves in the surgical field and electrophysiological recording of the target organs’ response. Development of intraoperative neuromonitoring methods for autonomic nerves bears challenges because known neuromonitoring techniques like electromyography (EMG) and stimulation and recording of evoked potentials (EP) are limited to the motor and sensory nerve system, which differs in excitation and stimulus response behaviors from the autonomic nerve system. Autonomic nerve stimulation does not result in stimulus-synchronous compound muscle action potentials but leads to a modulation of smooth muscle activity by evocation of action potential salvos (spikes). Spikes are triggered when physiologically spontaneous rhythmic changes of the membrane potential exceed a threshold potential, which leads to a slow contraction of the smooth muscle for several seconds^15,16^. This slow contraction of the smooth muscle occurs in the scope of intraoperative neuromonitoring after applying stimulation pulses to nerve tissue^17,18^.

The only pelvic neuromonitoring method on the market has been investigated and presented in studies by the research group Kauff and Kneist et al. The method is based on direct pelvic nerve stimulation in the surgical field, EMG measurement on the internal anal sphincter (IAS) and bladder manometry. The EMG method was adapted to optimize EMG signal amplification and signal processing for detection of autonomic biosignals. An increased EMG activity on the IAS during direct stimulation of functional nerves is rated as a positive signal response. Bladder response is measured by increased intravesical pressure, which requires bladder filling with Ringer’s solution for each stimulation period^5,19–21^.

This study presents a new approach to intraoperative pelvic neuromonitoring based on bioimpedance measurement, which is expected to allow rapid detection of pelvic autonomic nerves without the need for bladder filling as well as easy and assisted signal interpretation. The method consists of direct nerve stimulation in the surgical field and impedance measurement on the target organs, the urinary bladder and rectum. A change in tissue impedance on the bladder and/ or the rectum compared to the status before nerve stimulation is rated as an indicator of smooth muscle contraction and therefore identification of functional nerves. The technical feasibility of the method was proven in a preclinical animal study with twelve female pigs^17^.

The objective of this study was to transfer the method to a clinical setting for rectal surgery and to outline clinical feasibility. In addition, the study aimed to extract bioimpedance signal characteristics to compare positive signal responses that correlate with target organ contraction during stimulation of intact nerves and negative signal responses that correspond to stimulation of a control tissue portion.

Considering that the above-described pelvic neural network oversees the smooth muscle of the rectum and bladder, it's imperative to avoid conditions involving dysfunction of these organs, especially fecal and urinary incontinence, obstruction, or voiding issues. This means that the method should allow fibers of the hypogastric plexus innervating the rectum and detrusor muscle to be stimulated intraoperatively, identified and thus preserved.

## Methods

### Study design

The study was a prospective clinical investigation of 30 patients undergoing rectal resection for rectal cancer or diverticulitis, resection rectopexy for rectal prolapse or rectal extirpation for rectal cancer using an open, laparoscopic, or robotic approach. ASA 1–3 and age > 18 are additional inclusion criteria. Exclusion criteria are pregnant patients and patients with active implants, epilepsy, or severe cardiac arrhythmia as well as patients dependent on the sponsor. Informed consent was obtained from all participants or their legal guardians.

Each patient undergoes preoperative assessment of urinary and fecal function after radiotherapy, intraoperative use of pelvic neuromonitoring and assessment of urinary and fecal function up to one year postoperatively. To assess the urinary and fecal function, the residual urine is measured, and the patients are interviewed according to the International Prostate Symptom Score (IPSS) and Low Anterior Resection Syndrome Score (LARS). Primary endpoints of the study are the technical and clinical feasibility of the method.

The study was approved by the Ethics Committee of the Landesärztekammer Baden-Württemberg (Application No. 00011915/00054594) and the Higher Federal Authority (BfArM) (Application No. 94.1.12-5660-11914). It is registered in the German Clinical Trials Register DRKS00017437 (date of first registration 31/03/2020), accredited by the German Cancer Society (DKG) (Registry No. ST-D528) and conducted according to the Declaration of Helsinki and the DIN EN ISO 14155 at Ludwigsburg hospital.

### Intraoperative neuromonitoring technology

**Figure 1:**
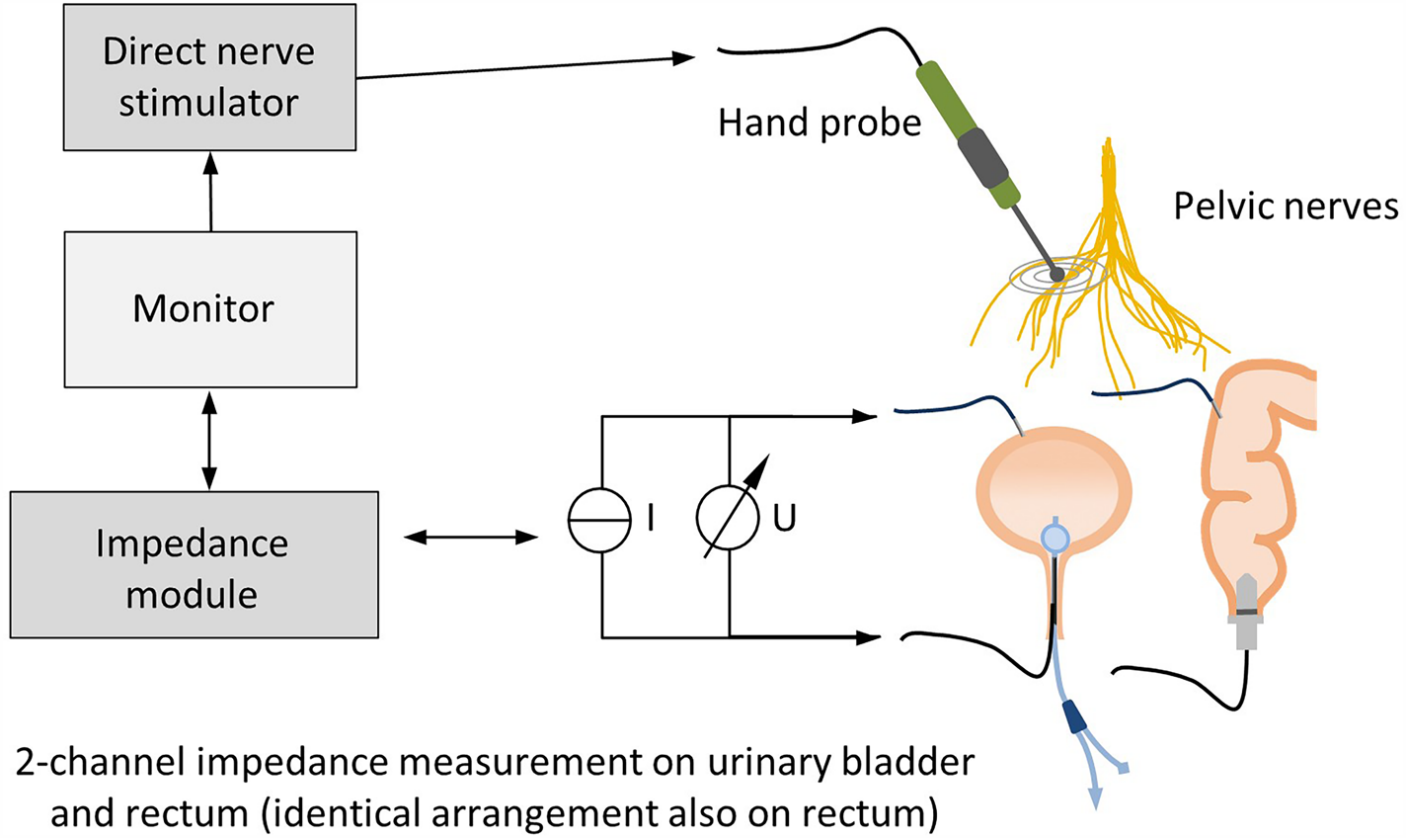
Schematic illustration of the neuromonitoring setup. Pelvic nerves are stimulated with a hand probe connected to a constant current stimulator. Target organ response is measured with impedance measurement, with two electrodes each being applied to the bladder and rectum. The impedance as a function of time is displayed on the neuromonitor.

The intraoperative setup consists of direct nerve stimulation in the surgical field and impedance measurement on the urinary bladder and rectum (schematic setup illustration see Fig. 1). The prototype of a new neuromonitoring system for pelvic autonomic nerve monitoring AVALANCHE® NeuroNeB (Dr. Langer Medical GmbH, Waldkirch, Germany) approved for this clinical investigation was used.

The preclinical animal study published in 2022 introduced impedance measurement as a new technology in intraoperative neuromonitoring including hardware design and analog filtering. In this method a test current (alternating constant current of 50 µA and 50 kHz) is applied to the tissue of each target organ via two electrodes. The voltage drop across the tissue is measured with a differential amplifier, amplified (gain of 50), high-pass filtered (cutoff frequency of 160 Hz), and lock-in amplified. By means of analog-to-digital conversion (ADC) the resulting direct current (DC) signal is converted to an output signal U(t), which represents a processed voltage drop. This processed voltage drop U(t) is displayed on the neuromonitor as a function of time, proportional to the target organs’ tissue impedance^17^. The application software of the neuromonitoring system stores the recorded raw data (processed voltage drop across the tissue U(t)) and the stimulation parameters in TDMS files (Technical Data Management Streaming, file format by National Instruments Corporation, Austin, USA) with a sampling rate of 10 Hz. No additional software signal processing is applied.

### Intraoperative neuromonitoring application

**Figure 2:**
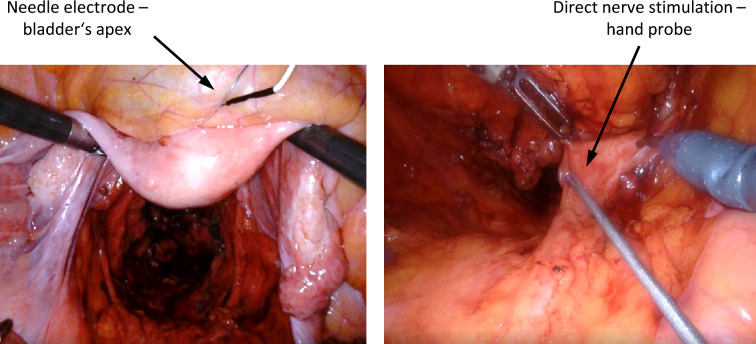
Intraoperative electrode setup during robotic TME. A needle electrode was placed in the bladder’s apex (left), direct nerve stimulation was performed with a bipolar laparoscopic hand probe (right).

For impedance measurement on two target organs, e.g. the bladder and rectum, two electrodes on each organ are needed, detecting the contractile tissue between the electrodes (schematic setup illustration see Fig. 1). A needle electrode (monopolar needle electrode, needle length 18 mm, needle diameter 0.4 mm, item no. MN4018D25S, Spes Medica S.r.l, Italy) was inserted at the bladder’s apex from the surgical site (see Fig. 2 left) and a catheter electrode (Disposable Urethral Catheter Electrode, size 14 Fr or 16 Fr, item no. UE002 or UE003, Spes Medica S.r.l, Italy) was positioned on the urethral sphincter. Thus, the tissue of the detrusor muscle between the needle electrode and urethral electrode is captured for impedance measurement.

At the rectum, a needle electrode (monopolar needle electrode, needle length 18 mm, needle diameter 0.4 mm, item no. MN4018D25S, Spes Medica S.r.l, Italy) was used in the upper rectum and a rectal probe (Probe Electrode with ring electrodes, electrode surface width 10 mm for males, item no. 61013A; electrode surface width 5 mm for females, item no. 61020A, Everyway Medical Instruments Co., Ltd.) was positioned in the anal canal. Therefore, the impedance of the tissue layer between the needle electrode in the upper rectum and the rectal probe in the anal canal is assessed when applying the monitoring.

Direct nerve stimulation was performed with a bipolar (reusable stimulation probe, tip diameter 0.5 mm per pole, item no. Sl2F0300M0032R, Spes Medica S.r.l, Italy) or monopolar (reusable stimulation probe, tip diameter 1.5 mm, 30° angled tip, item no. Sl1lC300M0032R, Spes Medica S.r.l, Italy) laparoscopic hand probe and a constant current stimulator that delivered monophasic square-wave pulses in the range of 10–20 mA, with 1000 μs pulse width, and a pulse frequency of 30 Hz (see Fig. 2 right).

During the surgical procedure the impedance on the urinary bladder and rectum was recorded continuously. Direct nerve stimulation was used when the surgeon intended selective localization of the superior or inferior hypogastric plexuses (left and right branches) during surgical preparation. The final measurement was taken exclusively on the urinary bladder after complete TME and transaction of the rectum by linear stapling, immediately before extraction of the specimen.

### Intraoperative signal interpretation

In order to evaluate the stimulation-induced muscle response, intraoperative signal interpretation and analysis was performed only during the direct nerve stimulation phases. It was investigated whether a stimulation-induced characteristic impedance change (positive signal response) occurred due to smooth muscle contraction, which is an indicator of the presence of functional nerves.

Criteria for evaluating an impedance change as a physiological stimulation-induced positive signal response are:

- new onset of change in the impedance signal after application of direct nerve stimulation,
- similarity of the signal waveform and morphology with the impedance signals derived in the animal study^17^,
- duration of the impedance change of several seconds, correlating with the duration of a slow smooth muscle contraction after evocation of spikes (3–15/min)^15,16^,
- confirmation of the positive signal response by a negative control in the surrounding tissue where no nerves are expected.

A negative signal response was considered as no change in the impedance signal after application of direct stimulation to a control tissue portion (e.g. wound margin, fat tissue). The impedance signal has been evaluated as unchanged if the absolute impedance level was the same before, during, and after direct nerve stimulation, based on the intraoperative, qualitative assessment of an application specialist. Random impedance changes caused by movements of the tissue whose onset did not coincide with the onset of stimulation and that differed from the expected waveform were considered as artifacts.

### Offline signal processing and analysis

**Figure 3:**
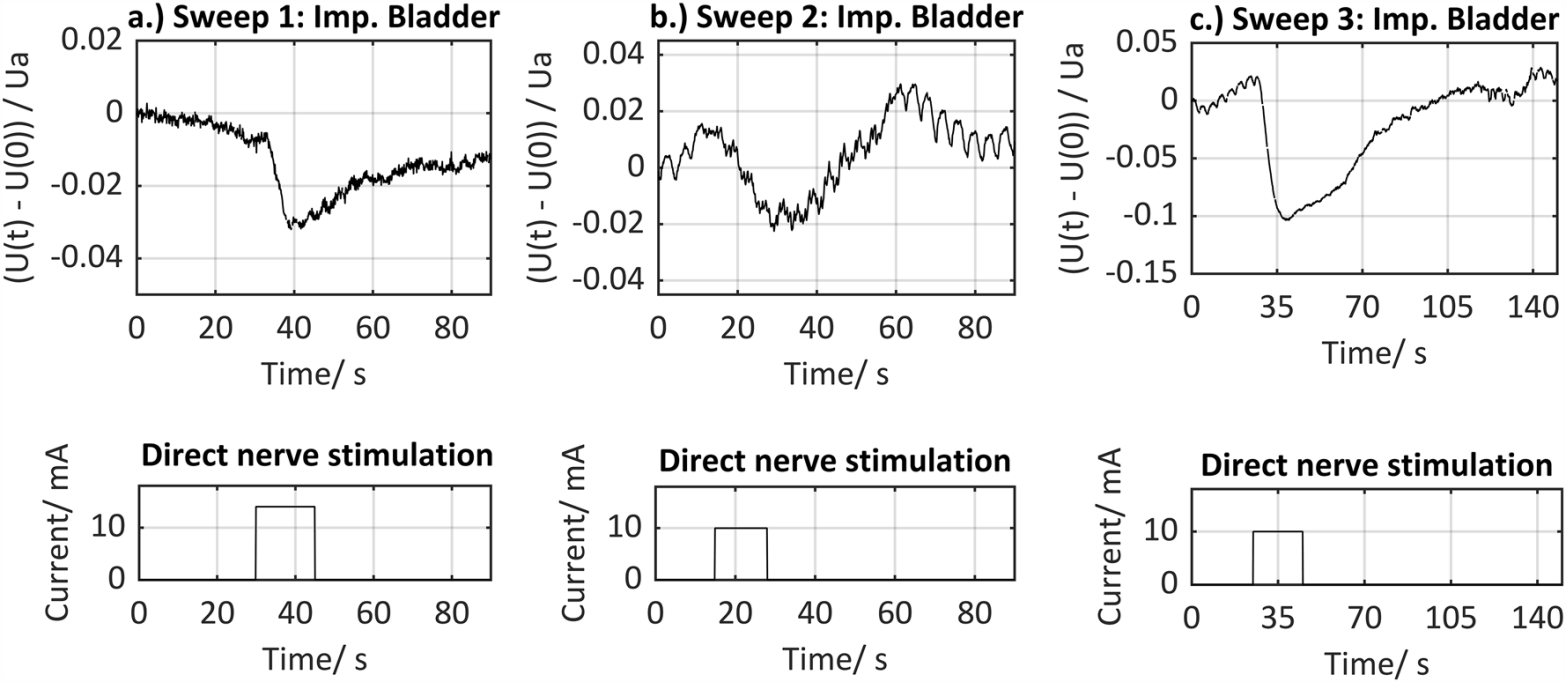
Examples of extracted signal sweeps containing a positive signal response on the urinary bladder (tissue impedance upper row, corresponding stimulation phases lower row). Signal characteristics especially the maximum amplitude and gradient can vary during intermittent stimulations.

Signal segments (hereafter referred to as signal sweeps) containing the direct nerve stimulation phases, including the signal responses analysed intraoperatively, were extracted from the acquired raw data. This resulted in two data sets containing signal sweeps with positive signal responses and signal sweeps with negative signal responses. Examples of signal sweeps with positive signal responses on the urinary bladder are illustrated in Fig. 3. A signal sweep is defined as the period from the onset of stimulation to relaxation of the muscle after contraction, which corresponds to the impedance level before stimulation.

**Figure 4:**
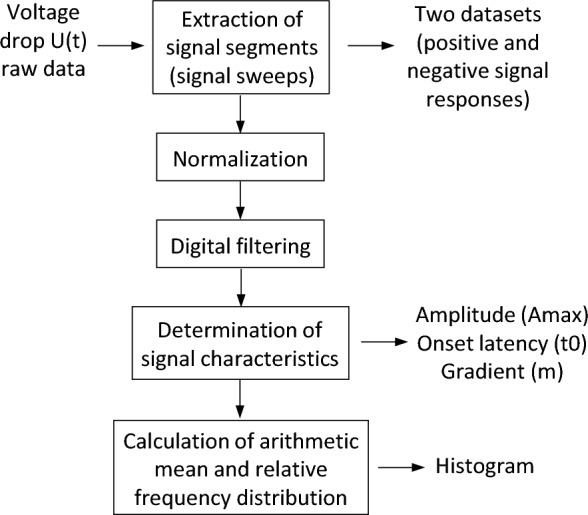
Flow chart of offline signal processing and analysis. Signal segments are extracted from the raw data, digital filtering is applied before the maximum Amplitude (Amax), the onset latency (t0) and the maximum gradient (m) are calculated and analyzed in a histogram.

Offline signal processing and analysis was applied exclusively to the extracted signal sweeps. It included signal normalization, digital filtering and calculation of signal characteristics using LabVIEW (National Instruments Corporation, Austin, USA), as can be seen in the flow chart in Fig. 4.

#### Signal normalization

Since the change in tissue impedance on the target organ is an indicator of smooth muscle contraction, the absolute tissue impedance value is not relevant. So, based on the results of the preclinical study, the signal sweeps were normalized to the basic impedance level Ua (impedance level before contraction)^17^.

#### Digital filtering

Low-pass filtering was performed to suppress artifacts of higher frequencies than the stimulation-induced signal, e.g. superimposed signals such as spontaneous changes in membrane potential (slow waves) or respiratory-caused artifacts.

#### Signal characteristics

**Figure 5:**
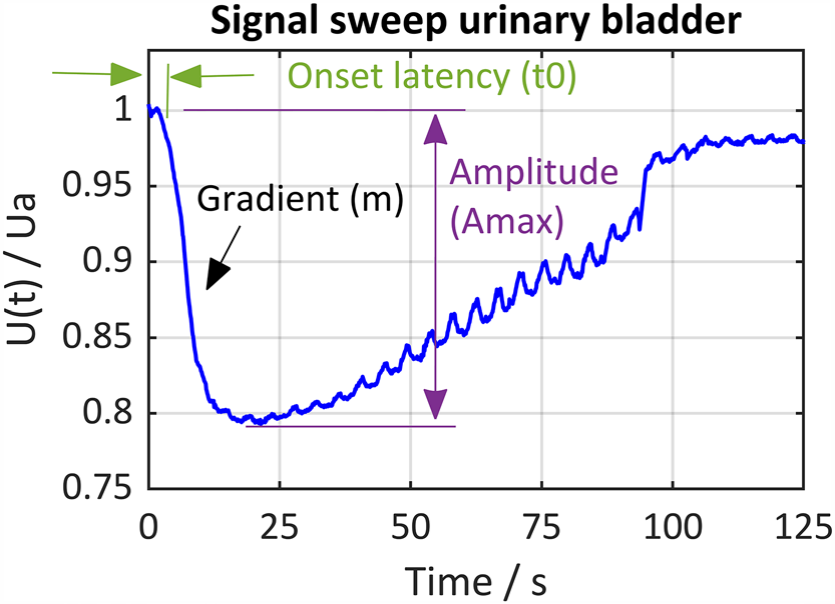
Illustration of the calculated signal characteristics within a signal sweep. Onset latency (t0), maximum gradient (m) and maximum amplitude (Amax).

For the normalized and filtered signal sweeps, the signal characteristics determined included the maximum amplitude (Amax) within the signal waveform, the onset latency (t0) of the impedance change, and the maximum gradient (m) within the signal waveform (illustration of the calculated signal characteristics, see Fig. 5). The arithmetic mean of the values within this signal characteristics, and the relative frequency distribution of the values were calculated and plotted using MATLAB® R2022b.

A high dispersion of the values in the histogram within a signal characteristic was evaluated as disadvantageous. The lower the dispersion of the values of a signal feature, the more specifically this parameter describes the signal and the more suitable this parameter is for the development of an algorithm.

### Statistical analysis

Sample size calculation was carried out with G*Power 3.1.9.2 based on the results of the preclinical animal study (type I error α = 0.05, type II error β = 0.2, power = 0.8).

As a comparison of the signal characteristics of the positively rated and the negatively rated signal responses is required, statistical analysis is based on metric measures. To compare the obtained maximum amplitudes (Amax) within the signal waveforms of the two data sets, the two-sided t-test was applied.

## Results

### Patient status at baseline

Of the 30 patients recruited, 26 underwent nerve- sparing TME in a robotic approach, three patients underwent an open approach, and one patient underwent a laparoscopic approach, with all using intraoperative pelvic neuromonitoring. TIVA was performed in 14 cases, gas anesthesia in 16 cases. Further investigations or comparisons of signal responses recorded with gas and intravenous anesthesia were not part of the current study. In all cases, muscle relaxants were administered and monitored using the train-of-four (TOF) method. This was done according to the usual anesthetic procedure in the hospital without the use of separate measures. Of the 30 patients, 16 were male (53.3%) and 14 were female (46.7%). Preoperatively, 12 of the 30 patients (40%) had no or mild urinary dysfunctions (IPSS score < 8 or residual urine < 20 ml), 17 patients (56.7%) had medium urinary dysfunctions (IPSS score 8–19 or residual urine 20–150 ml), and one patient (3.3%) had major preoperative urinary dysfunctions (IPSS score 20–35 or residual urine > 150 ml). 17 of the 30 patients (56.7%) had no fecal dysfunctions (no LARS, score 0–20), 6 patients (20%) had mild fecal dysfunctions (minor LARS, score 21–29), and 7 patients (23.3%) had major preoperative fecal dysfunctions (major LARS, score 30–42).

### Intraoperative neuromonitoring application

In 28 of the 30 cases (93.3%), the superior and/or inferior hypogastric plexus was initially iteratively identified (visually and using neuromonitoring) before dissection into the pelvic floor. Iterative stimulations were performed by repeatedly stimulating the same structures to verify the results and to identify the nerve course further. Thus, the method has proved clinical feasibility in 93.3% of the cases.

The mean number of iterative stimulations that resulted in a stimulation-induced positive signal response in these cases was 4 (range 2–12). In 26 of the 30 cases (86.7%), functional control of the identified superior and/or inferior hypogastric plexus was possible after complete TME and transaction of the rectum by linear stapling. The mean number of iterative stimulations for the final measurement that resulted in a stimulation-induced positive signal response in these cases was 3 (range 1–6). Stimulation of small bowel loops or surrounding tissue resulted in a negative signal response. This allowed pelvic autonomic nerves to be identified easily and directly and thus to be preserved. Impedance measurement on the urinary bladder was carried out on the empty bladder in all cases, which allowed fast identification of the innervating nerves. The time required for electrode placement and repeated performance of stimulation was less than 30 min per case. Electrode placement could be well integrated into the intraoperative workflow. The application of needle electrodes into the bladder’s apex and the upper rectum was possible and accessible from the surgical site. In laparoscopic and robotic procedures, the outlet of electrode cables was feasible but had the disadvantage that cable routing may interfere with instrumentation. In addition, in some cases, needle electrodes dislocated from the target muscle during the procedure. Although repositioning requires an additional step, it is not critical with respect to the evaluation of the derived signals during the case, because no quantitative comparison of the signals acquired within a single procedure was performed, which would have required unchanged electrode placements during the whole procedure.

Figure 3 shows three positive signal response examples with corresponding stimulation phases. The signal characteristics, in particular the maximum amplitude and the gradient, can vary intra- and interindividually depending on intermittent stimulation phases.

### Intraoperative signal interpretation

Intraoperative signal interpretation resulted in 170 positive signal responses at the urinary bladder and 69 positive signal responses at the rectum from 30 surgeries (239 positive signal responses in total), which were extracted from the raw data as described above. Negative signal responses were compared with the positive signal responses.

### Offline signal processing and analysis

Normalization of the extracted signal sweeps was accomplished by dividing the value of each sample U(t) by the basic impedance level Ua. Ua corresponds to the impedance of the device–wires–electrode–tissue connection and is determined by calculating the mean of the first fifteen samples after stimulation onset. After baseline correction by subtracting U(0)/Ua, a dimensionless signal sweep with an onset equal to zero (U(t) − U(0))/Ua) was obtained, indicating the change in tissue impedance during muscle contraction.

For digital low-pass filtering, a 3^rd^ order infinite impulse response bessel filter (IIR filter) with a cutoff frequency of 0.15 Hz was implemented, which proved to be suitable for filtering the signal in a way that facilitates the automatic calculation of the amplitude and latencies of the stimulation-induced signal without changing the signal characteristics significantly. Using an IIR filter instead of an FIR filter (finite impulse response filter) is advantageous because an IIR filter with comparable amplitude response can be realized with significantly lower order, which is essential with regard to a planned intraoperative online signal processing method^22^. To overcome the effect of non-linear phase response in the IIR filter, backward filtering was performed, resulting in a near-zero phase filter^22^.

The raw signal sweeps, the normalized and filtered signal sweeps, and the determined first derivative and integral of the filtered signal sweeps were used to calculate the signal characteristics. The maximum amplitude (Amax) of the impedance change within a signal waveform was calculated in percent by peak detection in the filtered signal, with the detection threshold set at 25% of the maximum value of the sweep. Depending on the result of the calculated integral value, it is essential to search for peaks or valleys as the impedance change can be an increase (upslope) or decrease (downslope). To assess the onset latency (t0) of the characteristic signal, peak detection of the first derivative of the signal was used to determine the first maximum representing the gradient (m) of the signal. Starting inversely from this point of time, the zero-crossing in the raw signal corresponding to the onset latency of the impedance change was identified.

The mean value of the maximum amplitudes (Amax) of the positive signal responses on the urinary bladder carried out from 170 signal sweeps is Amax = 3.3%, the mean onset latency of the positive signal responses is t0 = 2.3 s, and the mean value of the maximum gradients is m = 0.5%/s. On the rectum, the amplitudes and latencies were calculated from 69 signal sweeps and resulted in similar values: Amax = 5.2%, t0 = 2.3 s, and m = 0.8%/s.

**Figure 6:**
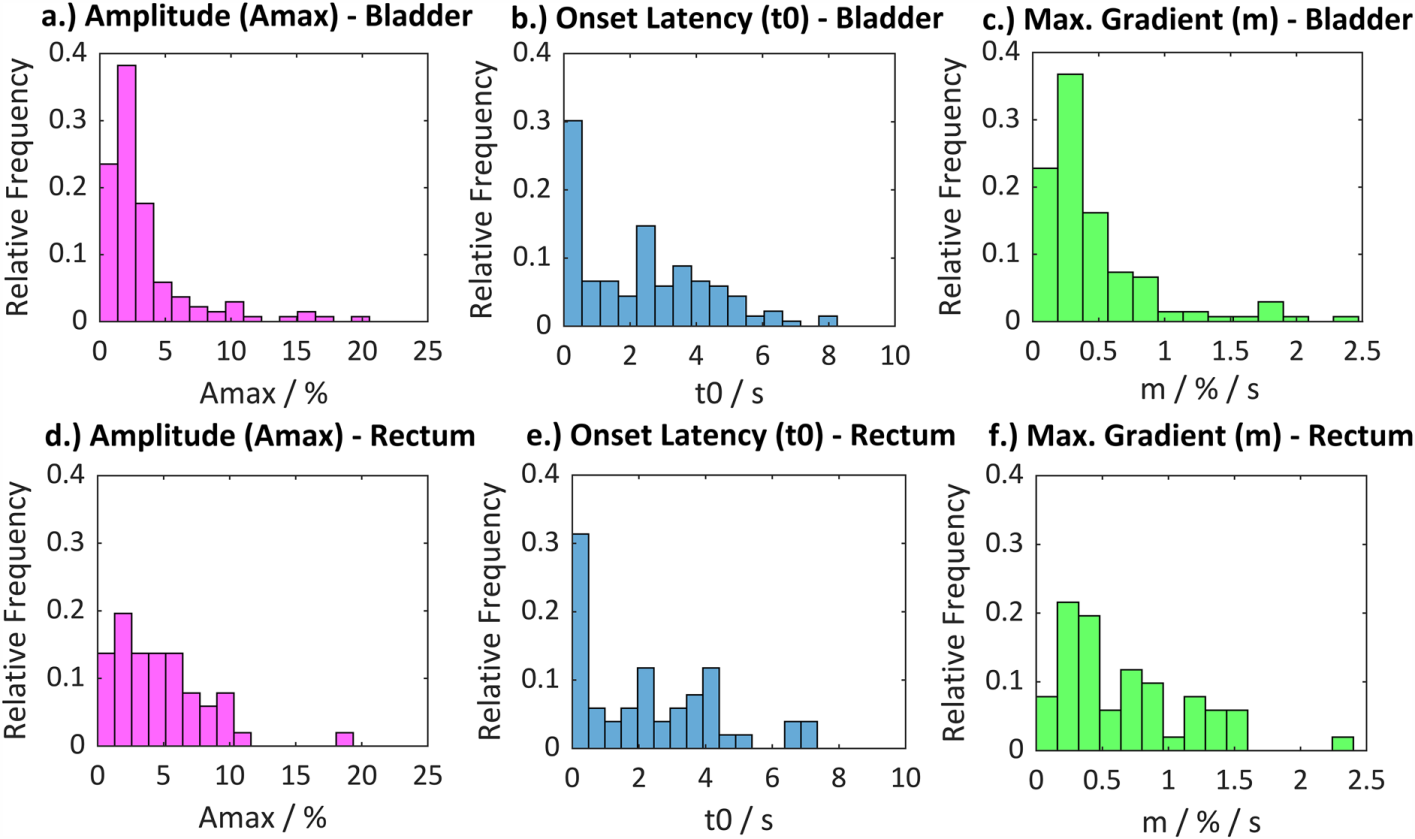
Relative distribution of values for onset latency t0 in seconds (left), maximum impedance change Amax in percent (middle), and maximum gradient m in percent/second (right). The relative frequencies were determined from 170 signal sweeps at the urinary bladder (n = 170, number of histogram bins = 15) and from 69 signal sweeps at the rectum (n = 69, number of histogram bins = 15).

Different excitation and smooth muscle contraction behaviors result in different signal morphologies, affecting signal amplitude and gradient. Figure 6 shows the relative frequency distribution of the calculated maximum amplitudes, gradients, and onset latencies on the urinary bladder and the rectum.

The onset latency shows a high dispersion of the values on both target organs, indicating varying time to reach the threshold for smooth muscle excitation. This is due to the different numbers of reached nerve fibers, varying distance of the stimulation site to the nerves, and varying amount of surrounding tissue, as well as hand probe movement during stimulation. The majority of the positive responses on the bladder and rectum have an onset latency of < 0.5 s. Due to the relatively high dispersion of the values, these values are covered within one histogram bin (0–0.5 s), but are unequal to 0 s. Positive responses with a comparatively short onset latency were detected during stimulation of nerve fibers, which were surgically exposed, where the hand probe could be applied directly to the nerves.

The relative frequency distributions of the maximum amplitude and gradient values are similar and have a lower dispersion than the onset latency. There is no significant difference between the signal characteristics on the urinary bladder compared to the rectum, therefore the signals are summarized for statistical analysis.

Amplitudes > 5% and gradients > 1%/s are less frequent, but may occur in rare situations such as during stimulation of main nerve branches (e.g. superior hypogastric plexus) with a high degree of exposure. The mean value of the maximum gradients of 0.5%/s implies a low-frequency impedance change, which is in accordance with the neurophysiological characteristics of smooth muscle behavior.

The negatively rated signal responses with no stimulation-induced impedance changes have an amplitude variation of 0.4% on average with a mean value of maximum gradients of 0.01%/s.

### Statistical analysis

The paired two-sided t-test was used to evaluate whether the mean maximum amplitude (µ1 = 3.8%, sd = 3.5%) of the positively rated signal responses obtained intraoperatively from both target organs (n = 239 signals) differ significantly from the mean amplitude variation in the negative signal responses (µ2 = 0.4%):

$$Null \,  hypothesis H0= \mu 1-\mu 2=0$$

$$Alternative \, hypothesis H1= \mu 1-\mu 2\ne 0$$

$$T=\frac{d}{sd*\frac{1}{\sqrt{n}}}= \frac{\left(3.8-0.4\right)\%}{3.5 \%*\frac{1}{\sqrt{239}}}= \mathrm{15,02}$$

$$t\left(1-\frac{\alpha }{2}, n-1\right)=1.972$$

$$H0 \, rejection \left(-\infty ,-t\right] \cup [t,\infty )$$

with T = Test statistic, t = test value, d = difference of the mean values and sd = standard deviation.

As $\infty >T>t$, the null hypothesis H0 can be rejected. Thus, the maximum amplitudes are significantly different for positive and negative signal responses.

### Patient outcome

**Figure 7:**
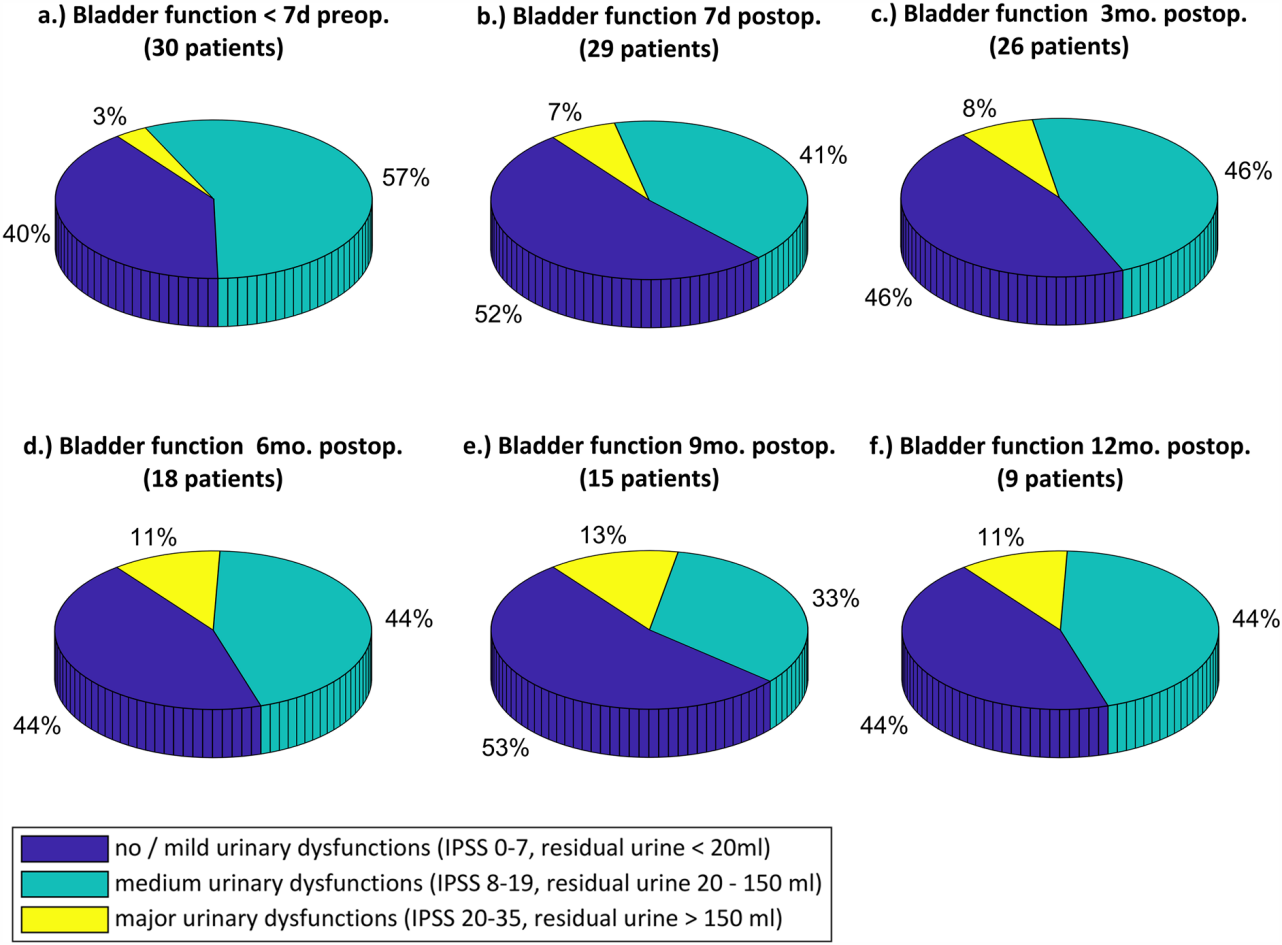
Bladder function of patients based on IPSS and residual urine volume. Bladder function was assessed < 7 days preoperatively, 7 days postoperatively, and 3, 6, 9, and 12 months postoperatively. Bladder function of the patients can be assessed as equivalent pre- and postoperatively.

Figure 7 shows the bladder function of the patients both before and after surgery. The percentage of patients who had no or mild urinary dysfunctions is slightly higher in the postoperative surveys at 44–53% than preoperatively at 40% of patients. Preservation of bladder function was thus demonstrated. The number of patients with medium symptoms decreased slightly postoperatively with 33% to 46% of patients compared with 57% of patients preoperatively, while the proportion of patients with major urinary dysfunctions increased slightly. However, when assessing outcome, it must be taken into account that the follow-up period has not been completed for all patients at present. For example, when urinary bladder function was determined at twelve months postoperatively, 11% of nine patients with severe bladder dysfunction corresponded to one patient. Identically, only one patient had severe symptoms preoperatively (3% of 30 patients). Furthermore, because the patient survey is based on subjective feelings of the patients, there may be day-dependent differences in the repeated surveys. In summary, the bladder function of the patients can be assessed as equivalent pre- and postoperatively. From the results, it can be concluded that autonomic nerve function was preserved, thus providing initial evidence of the clinical benefit and clinical safety of the method.

The functional outcome on rectal function cannot be adequately assessed at present. Rectal function cannot be determined until the stoma has been removed and even then, cannot be attributed exclusively to nerve function. The resection level as well as the type of anastomosis performed, further influence postoperative bowel function. Seven patients in the study could currently already be questioned about bowel function. Of these, two patients (29%) had no bowel function disorders (no LARS, score 0–20), another two patients (29%) had mild symptoms (minor LARS, score 21–29), and three patients (43%) had severe symptoms (major LARS, score 30–42). Therefore, the small number of patients interviewed does not allow a sufficient assessment of the outcome at this time.

## Discussion

The current results of the clinical investigation demonstrate that impedance measurement at the urinary bladder and rectum together with direct stimulation of the pelvic autonomic nerves is a feasible and promising method for intraoperative pelvic neuromonitoring. Contraction of the bladder and/or rectuminduced by direct stimulation of the innervating autonomic nerves resulted in a change in the bioimpedance of the tissue between one electrode on the caudal part and a second electrode on the cranial part of the organ. Technical and clinical feasibility was confirmed for open, laparoscopic, and robotic surgery. The method enabled intraoperative localization of the superior and inferior hypogastric plexuses and hypogastric nerves, including the left and right branches allowing the nerves to be preserved by the surgeon.

The tissue impedance measurement is a new technology for intraoperative electrophysiological recording of the smooth muscle response elicited by autonomic nerve stimulation. Tissue impedance is displayed on the neuromonitor as a function of time and can be interpreted intraoperatively by the surgeon.

Out of a total of 30 patients, nerve localization by impedance measurement could be performed in 26 patients before and after complete TME and transaction of the rectum by linear stapling. In two of the remaining four patients a positive signal response could be detected before but not after dissection and stapling. In these two cases, the positive signal responses were weak and difficult to interpret from the beginning of the surgery. Difficult interpretation can be attributed to the appearance of low signal amplitudes and artifacts occurring before and after the stimulation period. Additionally, one of these patients suffered from multiple sclerosis, which may have influenced neuromonitoring in general^23^.

During surgery in the two cases where no positive signal response could be detected at all, the following problems occurred:

*Case 1*: Repeated problems with gas loss when the stimulation probe was guided during the laparoscopic robotic procedure, so that even after troubleshooting neuromonitoring could not be performed as a standard procedure.

*Case 2:* Open procedure with unexpected high tumor location on opening. A sigmoid resection was performed, and additionally, an unexpectedly high metastatic involvement was detected, including the liver. This required focusing on performing multiple resections, thus neuromonitoring could only be used after resection. The surgical site was very moist throughout the abdomen, which may have influenced the derivability of muscle response by impedance measurement as the testing current required for impedance measurement may have been short-circuited across the fluid.

A moist stimulation position during direct nerve stimulation can also lead to false-negative responses. It is inherent in the direct nerve stimulation method that electrical currents in the tissue split according to Kirchhoff's law depending on the electrical resistance. Due to fluid accumulation in monopolar stimulation the current can also take other paths from the stimulation probe to the reference electrode without reaching the excitable cells of the nerve to be identified. In the case of bipolar stimulation fluid accumulation in the stimulation area can lead to short-circuiting of the poles, and the cells to be excited are not reached either. This behavior cannot be measured by the device since only the total flowing current can be determined. The literature on international standards and guidelines for electrophysiological monitoring also describes this behavior of electrical nerve stimulators, specifying that blood or fascia surrounding the nerve may cause inadequate current delivery and thus a false-negative stimulation response^24^.

In applying the method, another problem was observed. The positioning of the needle electrode in the bladder has a major impact on the detectable signal response. Since identification of the bladder’s apex can be difficult in adverse anatomical situations, the contractile muscle section may not be completely covered by the two measuring electrodes, and the change in tissue impedance may not be detectable or may result in a weak amplitude even though the muscles have contracted. Blocking the bladder catheter during the surgical setup until the first electrode positioning can help identify the apex of the bladder as the bladder is filled with urine. After electrode positioning the bladder can be emptied.

The applied offline signal processing and analysis should be performed intraoperatively in a market-ready system in near-real time to provide the surgeon with feedback on the stimulation response as early as possible. Digital low-pass filtering by an IIR-filter to suppress higher-frequency artifacts such as respiratory-caused signals proved to be an effective tool for smoothing the signal without changing the signal characteristics significantly but allowing automatic calculation of the amplitude and latencies of the stimulation-induced signal.

An algorithm for automatic detection of stimulation-induced positive signal responses needs to be applied only to direct nerve stimulation phases. To develop the algorithm, signal characteristics that specifically describe the physiological signal waveform must be identified and summarized in a feature vector. As the onset latency (t0) varies due to differences in the number of nerve fibers reached, the distance to the nerves, and the amount of surrounding tissue, as well as the movement of the hand probe during stimulation, this parameter may not be suitable for creating a feature vector. However, physiological signals may be characterized by a defined threshold and range of the maximum amplitude (Amax) and/ or a maximum gradient (m) since both signal characteristics differ significantly between positive and negative signal responses.

Automatic detection of positive signal responses requires discrimination of both negative signal responses and artifacts. As this study does not include the determination of signal characteristics that allow discrimination of artifacts, further signal features that additionally describe the signal shape and morphology of the stimulation-induced impedance change must be identified. This could include signal characteristics in time and time–frequency domain. An implemented algorithm for the automatic detection of stimulation-induced positive signal responses including discrimination from artifacts could assist the surgeon in signal interpretation, especially since this is a new application in medical technology that requires experience in recognizing the characteristic signal shape.

Small sample size and the heterogeneous patient population including rectal cancer, diverticulitis, and rectal prolapse associated with various preoperative and postoperative functional disorders depict limitations of the presented study. As the objective of the presented study was transfer a new approach to intraoperative pelvic neuromonitoring based on bioimpedance measurement to a clinical setting for rectal surgery, the study focused on determining clinical feasibility. Further studies to evaluate (long term) functional outcomes as well as sensitivity and specificity of nerve identification using this method need to be performed. Other factors that may affect the result of nerve identification, such as patients with high BMI, should also be further investigated.

Pelvic neuromonitoring can be performed with known standard neuromonitoring methods such as transcranial motor evoked potentials (tcMEP) from the external urethral sphincter (EUS) and external anal sphincter (EAS), free-running and triggered EMG from the EUS and EAS as well as bulbocavernosus reflex (BCR) measurement and pudendal somatosensory evoked potentials (SSEPs). All of these methods monitor afferent and efferent fibers of the pudendal nerve that innervate the motor sphincter muscles in the pelvic floor, which is mandatory in sacral spinal procedures^25–28^. Functional control and identification of pelvic autonomic nerves are not covered, which is essential during low anterior resections.

A similar approach to impedance measurement and direct nerve stimulation is adopted by the method developed by the research group Kauff and Kneist et al. including direct pelvic nerve stimulation and EMG measurement on the IAS together with bladder manometry. Clinical benefit of pelvic intraoperative neuromonitoring utilizing this method could be evidenced within a recently published multicenter, randomized, controlled clinical trial, which outlines the importance of the topic^5^. A detailed comparison of the two methods is shown in Table 1. The EMG needle electrodes require an exact positioning in the IAS muscle, which can be done with endosonography. For impedance measurement it is sufficient that the muscle lies between the two measurement electrodes, which saves application time. EMG measurement is made on the IAS but not on the urinary bladder. The bladder is monitored with manometry, so that the bladder contraction causes an increase in intravesical pressure. Therefore, every intermittent stimulation period requires bladder filling with ringer’s solution, otherwise bladder contraction is not detectable^19,20,29^. By contrast, impedance measurement on the bladder does not require bladder filling so that contraction of a filled as well as an empty bladder always results in a tissue impedance change. Thus, tissue impedance displayed as a function of time has turned out to be a direct and easily interpretable indicator of the activity of the urinary bladder and the rectum.

**Table 1:** Comparison of methods: EMG measurement + bladder manometry and impedance measurement.

	EMG and manometry (Kauff and Kneist et al.)	Impedance measurement (present study)
Method	IAS/rectum: electric (EMG) Bladder: manometric (pressure)	Rectum: electric (impedance) Bladder: electric (impedance)
Applied sensors	IAS/rectum: needle or surface electrodes Bladder: Luer-lock connection + pressure transducer	Rectum: needle electrode + rectal probe Bladder: Needle electrode + urethral catheter electrode
Time effort application	Endosonography—if needles are used for IAS, Luer-Lock connection required)	Rectal probe + urethral catheter positioning as well as needle electrode positioning without technical assistance
Invasiveness	Can be used non-invasively	Invasive needle electrodes required
Bladder filling	Required for every stimulation period	No bladder filling required
Sensor wires in surgical site	No wires present in the surgical site	Two wires present in the surgical site
Signal interpretation/applicability bladder	IAS/ rectum: Interpretation of EMG activity using frequency analysis Bladder: intravesical pressure	Rectum and bladder: impedance change; direct, and easily interpretable; applicable for both rectum and bladder
Clinical benefit/patient outcome	Outlined in multicenter, randomized, controlled clinical trial	Pending

A different approach to pelvic neuromonitoring is intraoperative real-time imaging of nerves for identification and visualization. In 2017 Zhang et al. published a pilot study on intraoperative nerve staining with modified leucomethylene blue (MLB) including ten patients with cervical cancer during nerve-sparing radical hysterectomy. The authors reported that the minor nerves were dyed blue clearly, improving the systematic preservation of pelvic autonomic nerves further. However, they also reported major limitations of this method such as an insufficient penetration of the MLB and inadequate specificity^30^. Boyette et. al published their results of in-vivo imaging of cavernous nerves in male rats in 2007. The method required an injection of a fluorescent nerve tracer in the corpus cavernosum and imaging using fibreoptic confocal fluorescent microscopy. It could be used for nerve preservation during radical retropubic prostatectomy in the future, but the transfer to human medicine is still pending^31^. Another animal study with mice and rats was conducted by Hingorani et al. in 2018. They analysed the binding of the dye-labelled peptide FAM-HNP401 to nerves including autonomic nerves isolated from human prostate. The translation into a clinical setting for intraoperative identification is also pending^32^. It has been found that although real-time imaging of nerves does not provide information about the functionality of the nerves of interest, it could be a useful adjunct to functional intraoperative neuromonitoring.

## Conclusion

The new approach to intraoperative pelvic neuromonitoring consisting of direct pelvic nerve stimulation and impedance measurement on the urinary bladder and rectum was transferred to a clinical setting based on a preclinical animal study. Regarding technical aspects, the method was found to be a reliable method for a fast identification of hardly visible pelvic nerves. Clinical evaluation of long-term patient outcomes is pending, as the study is ongoing, and the postoperative patient survey is still open for most patients.

## Data Availability

All data generated or analysed during this study are included in this published article.
